# Fear of Massive Deportations in the United States: Social Implications on Deprived Pediatric Communities

**DOI:** 10.3389/fped.2017.00177

**Published:** 2017-08-21

**Authors:** Marie Leiner, Izul De la Vega, Bert Johansson

**Affiliations:** ^1^Pediatrics, Texas Tech University Health Sciences Center, El Paso, TX, United States; ^2^Political Science, UCLA, Los Angeles, CA, United States; ^3^El Paso Children’s Hospital, El Paso, TX, United States

**Keywords:** child development, poverty, executive orders, mental health services, massive hepatic necrosis, deportations, social effects

Inequalities in children’s well-being exist in both developed and undeveloped countries worldwide (1). Childhood experiences have a profound effect not only on children’s current lives but also on their future opportunities and prospects, especially for those who are at a disadvantage. These disadvantages can create an unstable environment for children with long-lasting effects (2).

Government agencies, medical societies, for profit and non-profit organizations, academics, and other groups have proposed different strategies to minimize the risks confronted by disadvantaged children but have only obtained moderate results. The success of these strategies depends on how a country’s government respects and supports the rights of children, a concept stated very clearly in Article 3 of the 1989 United Nations Convention on the Rights of the Child: “in all actions concerning children, whether undertaken by public or private social welfare institutions, courts of law, administrative authorities or legislative bodies, the best interests of the child shall be a primary consideration.” Therefore, economic, political, or environmental decisions at all levels must consider the pyramidal effect that these decisions will have on children’s development and well-being (3).

With the aforementioned in mind, political decisions in the United States must consider the impact of immigration policies on deprived pediatric communities both at the individual and collective level.

Within these communities, parents adopt negative coping mechanisms in response to severe stress and depression, which has a detrimental effect on their children and the family overall. As a result, many children will lack the essential human and social experiences that are needed to thrive and grow into healthy, productive adults. Any external forces applied to these deprived communities have a greater effect on children due to their developmental stage. In fact, studies suggest that the environment can play an important role in how the genetic code is expressed. For example, it has been found that living in poverty affects the brain development of children, with neurocognitive outcomes that include decreased reading/language ability and executive functions (4). Children living in poverty are more likely to experience chronic stressors that, without the presence of buffering relationships, can disrupt the development of brain architecture. Abuse during early childhood/adolescence is associated with subsequent memory problems and a risk for posttraumatic stress disorder. Children living in poverty are more likely to have a diminished frontal cortex volume, which has been implicated with behavioral, cognitive, and emotional problems (5, 6). The fear of massive deportations compounds stress in deprived communities resulting in different effects on children and adults (7, 8). Children will be the most affected whether they are living legally or illegally in the country. Children will struggle to comprehend the complexities of immigration policies that can result in the deportation of their neighbors, friends, or one or both of their parents. This news will create an environment of fear within the home, neighborhood, and the community at large, compromising the children’s fundamental right to safety.

Living in fear, as a global effect, can paralyze society but has an even greater effect among groups confronting disparities. These groups will experience limited access to the pillars that sustain society, including access to education, protection by the law, basic needs (e.g., food and housing, health care) and opportunities to plan for the future (9). For example, some parents who fear deportation might stop taking their children to school. As a result, these children will fall behind their peers. Reporting family abuse may be reduced because of the fear of having one or both parents deported, even though families have been, and are still currently, protected regardless of their legal status. Allowing the devastating effects of abuse on the lives of children to run unchecked will generate physical and psychological consequences that will cause life-long scars (10).

Fear of being detained might reduce opportunities for a family to obtain food and housing. This will leave them in limbo and unable to satisfy the most basic needs to survive. In the case of children, this will inhibit their growth and affect their brain development. Both preventative and urgent care will be put at risk when parents avoid seeking care. Children require early routine preventive care to detect developmental delays or problems; this has been established to encourage early interventions, which predict better outcomes. Making plans for the future is not feasible when living with uncertainty. It is essential for children to adapt and find their own identity, as well as their role in society. Children/adolescents who miss the opportunity to plan for their future often become victims of many physical, mental, and emotional problems (11).

Although the long-term effects of massive deportations on children have not been studied, exposure to similar conditions of massive or generalized fear [e.g., the threat of deportation (12–14), collective violence due to terrorism, wars, guerillas, and organized crime (15, 16)] has adverse outcomes and unpredictable social implications. The common effect of “living in fear” found in these studies is the most likely trigger for the negative outcomes, as well as a sense that society has failed to protect its citizens. The feeling that society has failed individuals is the seed that generates individuals who are dedicated to crime, delinquency, or who are simply disconnected from society and have no intention to positively contribute to a harmonious and balanced society (17).

Therefore, those writing these executive orders should consider that failure to obtain sufficient information about possible outcomes may result in undesirable and unintended consequences (18).

Besides the additional negative effect in deprived communities, a global effect of these orders can trigger potential unintended consequences involving increased racial/ethnic discrimination, feelings of stigma, and possible lower tolerance of racial/ethnic diversity. Moreover, these decisions cannot be taken lightly and should be accompanied by programs that improve social impact. There is a lack of a multidimensional approach for planning, understanding and considering all social, economic, and cultural implications of these decisions. Investment in early childhood programs that focus on families as an inseparable nucleus is vital. These programs will lay the foundation for improved health and growth in areas that are sensitive to external influences during a child’s development.

It is our obligation as a society in this great country to protect children and improve the conditions in which they develop by not forgetting that these children did not ask to be here. The force resulting from the fear of massive deportations will not only affect non-citizen families but will also affect every person in our country. Negative forces applied to vulnerable groups in any society have historically and systematically been proven to generate pervasive negative effects on children/adolescents—that should not be ignored and will most likely have costly consequences (19).

## Author Contributions

ML, IV, and BJ make substantial contributions to conception and design of draft a final paper, based on their professional experience.

## Conflict of Interest Statement

The authors declare that the research was conducted in the absence of any commercial or financial relationships that could be construed as a potential conflict of interest.
